# The State of Long Non-Coding RNA Biology

**DOI:** 10.3390/ncrna4030017

**Published:** 2018-08-10

**Authors:** John S. Mattick

**Affiliations:** Garvan Institute of Medical Research, Sydney, NSW 2010, Australia; Green Templeton College, Oxford OX2 6HG, UK; Genomics England, London EC1M 6BQ, UK; j.mattick@garvan.org.au

**Keywords:** regulatory architecture, lncRNA structure–function, epigenetic plasticity, evolution

## Abstract

Transcriptomic studies have demonstrated that the vast majority of the genomes of mammals and other complex organisms is expressed in highly dynamic and cell-specific patterns to produce large numbers of intergenic, antisense and intronic long non-protein-coding RNAs (lncRNAs). Despite well characterized examples, their scaling with developmental complexity, and many demonstrations of their association with cellular processes, development and diseases, lncRNAs are still to be widely accepted as major players in gene regulation. This may reflect an underappreciation of the extent and precision of the epigenetic control of differentiation and development, where lncRNAs appear to have a central role, likely as organizational and guide molecules: most lncRNAs are nuclear-localized and chromatin-associated, with some involved in the formation of specialized subcellular domains. I suggest that a reassessment of the conceptual framework of genetic information and gene expression in the 4-dimensional ontogeny of spatially organized multicellular organisms is required. Together with this and further studies on their biology, the key challenges now are to determine the structure–function relationships of lncRNAs, which may be aided by emerging evidence of their modular structure, the role of RNA editing and modification in enabling epigenetic plasticity, and the role of RNA signaling in transgenerational inheritance of experience.

## 1. A Surprising New Entrant into the Repertoire of Expressed Genes

A role for RNA as regulatory molecules, rather than just as templates (mRNAs) and components of the machinery (ribosomal RNAs, transfer RNAs, spliceosomal RNAs) for the production of proteins, was established in the 1990s with the surprising discovery of the RNA interference (RNAi) and related pathways, which utilize small RNA guides to regulate mRNA stability and translation, and to control transposons [1,2,3,4,5,6,7].

At the same time, seminal long non-protein-coding RNAs (lncRNAs), involved notably in X inactivation (Xist) [8,9,10,11] and in genomic imprinting (H19) [12](later shown to play a role in cancer [13])were also discovered.

The subsequent revelation from high-throughput cDNA sequencing (RNAseq) in the 2000s that tens of thousands of long intronic, intergenic and antisense lncRNAs are transcribed from the genomes of mammals [14,15,16,17,18,19,20,21,22,23,24,25,26,27] and other complex organisms [28] was also surprising. The range of cellular non-coding RNAs may be underestimated by sequencing strategies that target polyadenylated RNA, as many are derived from retrotransposons [29,30], introns [24] and/or derived by processing longer transcripts [31,32], including circular lncRNAs [33,34]. Some lncRNAs are extremely long [35,36]. Indeed, it appears that the vast majority of the genomes of all organisms, irrespective of the proportion that is protein-coding, is transcribed, mainly to produce non-coding RNAs [28].

## 2. An Unwelcome Player

However, despite the RNAi precedent, and with few exceptions [37,38,39], the existence of these uncharacterized lncRNAs was initially ignored or dismissed as “transcriptional noise”. Not only was it unclear how they might fit into the existing conceptual framework of genetic information and gene regulation, assumed to be transacted by proteins acting in combinatoric fashion, their sheer number, if functional, threatened the primacy of this framework, which has long been an article of faith in molecular, cellular and developmental biology.

The possibility that lncRNAs might be functional also contradicted the widely held belief, dating from the late 1970s, that the intronic and ‘intergenic’ sequences from which they are transcribed, and which dominate the real estate of the mammalian genome, are largely evolutionary debris (‘junk’) [40], comprised of hangovers from the prebiotic assembly of “genes” [41] expanded by accumulation of retrotransposon parasites (“selfish DNA”) [42]. 

The idea that the most non-coding RNAs are noise from biologically inert regions of the genome was superficially bolstered by the observation that most are expressed at low levels and are generally less conserved than protein-coding sequences (although there are notable exceptions [43]) similar to ancient retrotransposon-derived sequences, assumed to be non-functional and evolving ‘neutrally’ [44]. This is a circular argument of dubious merit [45], since there is increasing evidence that retrotransposon-derived sequences have been exapted for various functions and coopted as mobile modules to alter the patterns of gene expression [30,45,46,47,48,49]. The comparison of the rate of divergence of an extant set of ancient repeats also does not include the (unknown) number that have diverged to the point of unrecognizability, which therefore underestimates the rate of their presumed neutral evolution, and the extent of evolutionary selection on the genome [45].

The (lack of a high) conservation argument also fails to take into account the fact that adaptive radiation occurs mainly by the relatively rapid evolution of the regulatory sequences under positive selection, that such sequences have quite different structure–function constraints to proteins, and that they are subject to rapid turnover [45,50,51]. Thus, many lncRNAs are likely to be lineage-specific. 

At this point it is important to remember that the metazoan proteome is remarkably static. Both the nematode and human genomes contain ~20,000 protein-coding genes, most of which are functionally orthologous, despite orders of magnitude difference in their developmental (and cognitive) complexity. By contrast, the proportion of the genome that is non-protein-coding, and the number and range of non-coding RNAs expressed therefrom, increases with developmental complexity [28,52], raising the obvious possibility that these sequences are responsible for specifying developmental complexity and phenotypic diversity.

## 3. Evidence of Long Non-Coding RNA Functionality

Indeed, there are many different rate classes of sequence evolution in mammals, indicating that at least 45% of the alignable regions of mammalian genomes are not evolving neutrally [53], with at least 18% of the mammalian genome conserved at the level of predicted RNA structure [54]. 

There are also many indices of lncRNA functionality [55,56], including conservation of promoters [16,57], regulation by canonical transcription factors [58,59], and chromatin signatures of active gene expression [57,59]. Moreover, lncRNA exons have been found to be more conserved than neutrally evolving ancestral repeat sequences, albeit at lower levels than protein-coding genes [57].

The case for lncRNA functionality is also supported by their dynamic expression patterns in differentiating cells and their highly specific spatial (including subcellular) localization [57,60,61,62], especially in the brain [63,64], which also explains their low abundance in RNAseq analyses of whole tissues [26]. Indeed, high-resolution analyses using RNA capture technologies have revealed an extraordinary diversity of lncRNAs, most of which are likely to be cell-specific, and which have yet to be catalogued or characterized [27,65] Perhaps the most intriguing are the 3’UTR-derived lncRNAs that are expressed separately from, and appear to convey differentiation signals independently of, their normally associated mRNAs [66,67].

There have been many studies examining lncRNA biology over the past decade (too many to reference here, but see http://www.lncrnadb.org [68,69], https://lncipedia.org [70] and http://www.noncode.org [71]) linking lncRNAs with cellular processes, including the formation of specialized subnuclear organelles [72,73,74], chromatin domains [75], regulation of splicing [76,77], enhancer action [78,79,80], and binding to chromatin-modifying proteins such as polycomb [81,82,83,84,85], trithorax [61] and Dnmt1 [86,87]. Some of these functions may not be mediated by the lncRNA itself, but through mechanisms associated with their biogenesis [88,89,90,91]. There are also many studies linking lncRNAs with differentiation and development [92,93], and with diseases, including coronary artery disease and diabetes [33,94,95], schizophrenia [96] and cancer (see e.g., [43,97,98]). 

Knockdown of lncRNAs by small interfering RNA (siRNA)-related methodologies frequently results in observable changes in cellular behavior or characteristics in culture [55]. On the other hand, chromosomal deletion of lncRNA sequences often do not show overt phenotypic consequences. For example, only 5 of 18 lncRNA mouse knockouts resulted in lethality or growth defects [99,100]. However, most phenotypic screens do not examine behavioral or cognitive effects. For example, deletion of the widely brain expressed non-coding RNA BC1 showed no developmental consequences [101], but later tests showed the mutant mice, although having normal brain morphology and no obvious neurological deficits, exhibited decreased exploratory ability and increased anxiety [102].

In this context, it is worth noting that deletion of a subset of the most highly conserved sequences in the mammalian genome, ultraconserved elements (UCEs) [103,104], which are surely functional on the evolutionary evidence, also did not result in obvious abnormalities [105], although a later study showed subtle neurological alterations [106].

While skeptics remain, the most likely interpretation is that the documented functional examples are emblematic of an army of regulatory RNAs that guide epigenetic trajectories and specify cell state during a very complex and precise developmental ontogeny—from a single fertilized cell to a mobile, cognizant adult—and that most of the human genome is devoted to this purpose [37,38,39,107,108,109,110,111,112]. Indeed, the proportion of the mammalian genome devoted to cognitive function, rather than body plan development, may be considerably underestimated, given the preponderance of lncRNA expression in the brain [63]. Not surprisingly then, many lncRNAs are primate-specific [57,113,114].

Indeed, the growing body of evidence is now leading to a general acceptance of the relevance of (many or most) lncRNAs to cell and developmental biology [92], and increasingly neurobiology [63,64,96,115,116,117], with the debate, such as it remains, shifted to the proportion of lncRNAs that may be biologically relevant. For me, the best indicator, although by no means proof, is their precise expression patterns [26], on which basis one can project that most are likely to be functional. 

If so, the current protein-centric framework for understanding the genetic programming of differentiation and development is incomplete, a legacy of the mechanical worldview that held sway at the birth of molecular biology. Reconsideration of this framework to incorporate not only proteins but also structural and regulatory RNAs [109,111,118,119] is overdue. 

## 4. Long Non-Coding RNA Structure–Function Relationships 

The most pressing challenges now are to determine the structure–function relationships in lncRNAs and to parse their functional repertoire. This should resolve lingering questions and place lncRNAs into an integrated conceptual framework, together with small regulatory RNAs, transcription factors and signaling pathways, among others, for understanding the decisional hierarchies that control the 4-dimensional ontogeny of complex multicellular organisms [119].

There is logic and experimental evidence to suggest that lncRNAs have a modular architecture, given their likely role as scaffolds and epigenetic guide molecules [26,82,120]. This is strengthened by a recent high depth sequencing study that found, unexpectedly and in contrast to the limited information that had been previously available [57], that the internal exons of lncRNAs are almost universally alternatively spliced [27], which clearly implies modularity. 

If this is correct, the establishment of the exon as the primary unit of lncRNA structure–function, combined with the observation of conservation of lncRNA structure [54] and the presence of structural orthologs around the genome [121], should provide a framework for determining which structural RNA modules associate with which effector proteins [121]. It is envisaged that such studies will lead to expanded structure–function databases [122,123,124] whereby specific protein (e.g., polycomb) binding domains in regulatory RNAs can be identified genome- and transcriptome-wide, and thereby the roles of and effector pathways for different lncRNAs and their alternatively spliced isoforms. It may be much harder, as exemplified by snoRNAs, to determine the RNA and DNA targets of lncRNAs, and which modules impart this function.

This framework should also allow parsing of the different types and roles of lncRNAs in establishing chromatin territories, enhancer looping, guidance of epigenetic modifier proteins that impose DNA and histone modifications, and the formation of subcellular domains, among others. 

In addition, while most lncRNAs are nuclear and associated with chromatin, some are cytoplasmically localized [57,63] with functions yet to be discovered. There is increasing evidence that RNAs are involved in the nucleation of liquid crystal domains in conjunction with disordered RNA-binding proteins [125], potentially an entirely new dimension of cell biology beyond that of the well-characterized membrane-bound organelles. High-resolution imaging will be required, along with high-resolution RNA sequencing, and oligonucleotide or antibody capture to dissect the components of the structures where lncRNAs are localized.

## 5. From Hard- to Soft-Wiring

A new and rapidly emerging frontier is the role of RNA editing [126,127] and RNA modification [128,129] in modulating RNA signaling pathways in response to developmental cues and environmental signals, which may lie at the heart of the epigenetic plasticity seen in physiological adaption, complex diseases such as cancer and diabetes, and brain function [130].

There is still much to do to understand the role of small RNAs, especially the ti/spliRNAs that are derived from transcription start sites and exonic borders [131,132], and fragments of tRNAs [133,134,135] and snoRNAs [136], some of which may function as miRNAs [137,138,139], as well as to decipher their evolutionary links and the regulatory networks in which they participate.

Finally, and most intriguingly, is the role of RNA in intercellular and transgenerational inheritance (soft-wired inheritance), for which there is not only evolutionary logic [140,141,142] but also increasing evidence [143,144,145,146,147,148,149]. The emerging picture is not (simply) of RNA as a transient intermediate between ‘gene’ and protein, but rather as the central computational engine of cell biology, differentiation and development, brain function and perhaps even evolution itself. Many textbooks may have to be rewritten once the full dimensions of regulatory RNA biology are revealed. 

## References

[1] Lee R.C., Feinbaum R.L., Ambros V. (1993). The *C. elegans* heterochronic gene *lin-4* encodes small RNAs with antisense complementarity to *lin-14*. Cell.

[2] Wightman B., Ha I., Ruvkun G. (1993). Posttranscriptional regulation of the heterochronic gene *lin-14* by *lin-4* mediates temporal pattern formation in *C. elegans*. Cell.

[3] Ha I., Wightman B., Ruvkun G. (1996). A bulged *lin-4/lin-14* RNA duplex is sufficient for *Caenorhabditis elegans lin-14* temporal gradient formation. Genes Dev..

[4] Reinhart B.J., Slack F.J., Basson M., Pasquinelli A.E., Bettinger J.C., Rougvie A.E., Horvitz H.R., Ruvkun G. (2000). The 21-nucleotide *let-7* RNA regulates developmental timing in *Caenorhabditis elegans*. Nature.

[5] Lau N.C., Lim L.P., Weinstein E.G., Bartel D.P. (2001). An abundant class of tiny RNAs with probable regulatory roles in *Caenorhabditis elegans*. Science.

[6] He L., Hannon G.J. (2004). MicroRNAs: Small RNAs with a big role in gene regulation. Nat. Rev. Genet..

[7] Girard A., Hannon G.J. (2008). Conserved themes in small-RNA-mediated transposon control. Trends Cell Biol..

[8] Brown C.J., Ballabio A., Rupert J.L., Lafreniere R.G., Grompe M., Tonlorenzi R., Willard H.F. (1991). A gene from the region of the human X inactivation centre is expressed exclusively from the inactive X chromosome. Nature.

[9] Brockdorff N., Ashworth A., Kay G.F., Cooper P., Smith S., McCabe V.M., Norris D.P., Penny G.D., Patel D., Rastan S. (1991). Conservation of position and exclusive expression of mouse *Xist* from the inactive X chromosome. Nature.

[10] Brockdorff N., Ashworth A., Kay G.F., McCabe V.M., Norris D.P., Cooper P.J., Swift S., Rastan S. (1992). The product of the mouse *Xist* gene is a 15 kb inactive X-specific transcript containing no conserved ORF and located in the nucleus. Cell.

[11] Brown C.J., Hendrich B.D., Rupert J.L., Lafreniere R.G., Xing Y., Lawrence J., Willard H.F. (1992). The human *XIST* gene: Analysis of a 17 kb inactive X-specific RNA that contains conserved repeats and is highly localized within the nucleus. Cell.

[12] Brannan C.I., Dees E.C., Ingram R.S., Tilghman S.M. (1990). The product of the *H19* gene may function as an RNA. Mol. Cell Biol..

[13] Raveh E., Matouk I.J., Gilon M., Hochberg A. (2015). The *H19* long non-coding RNA in cancer initiation, progression and metastasis—A proposed unifying theory. Mol. Cancer.

[14] Okazaki Y., Furuno M., Kasukawa T., Adachi J., Bono H., Kondo S., Nikaido I., Osato N., Saito R., Suzuki H. (2002). Analysis of the mouse transcriptome based on functional annotation of 60,770 full-length cDNAs. Nature.

[15] Rinn J.L., Euskirchen G., Bertone P., Martone R., Luscombe N.M., Hartman S., Harrison P.M., Nelson F.K., Miller P., Gerstein M. (2003). The transcriptional activity of human chromosome 22. Genes Dev..

[16] Carninci P., Kasukawa T., Katayama S., Gough J., Frith M.C., Maeda N., Oyama R., Ravasi T., Lenhard B., Wells C. (2005). The transcriptional landscape of the mammalian genome. Science.

[17] Katayama S., Tomaru Y., Kasukawa T., Waki K., Nakanishi M., Nakamura M., Nishida H., Yap C.C., Suzuki M., Kawai J. (2005). Antisense transcription in the mammalian transcriptome. Science.

[18] Cheng J., Kapranov P., Drenkow J., Dike S., Brubaker S., Patel S., Long J., Stern D., Tammana H., Helt G. (2005). Transcriptional maps of 10 human chromosomes at 5-nucleotide resolution. Science.

[19] Kapranov P., Drenkow J., Cheng J., Long J., Helt G., Dike S., Gingeras T.R. (2005). Examples of the complex architecture of the human transcriptome revealed by RACE and high-density tiling arrays. Genome Res..

[20] Birney E., Stamatoyannopoulos J.A., Dutta A., Guigo R., Gingeras T.R., Margulies E.H., Weng Z., Snyder M., Dermitzakis E.T., Thurman R.E. (2007). Identification and analysis of functional elements in 1% of the human genome by the ENCODE pilot project. Nature.

[21] Kapranov P., Cheng J., Dike S., Nix D.A., Duttagupta R., Willingham A.T., Stadler P.F., Hertel J., Hackermuller J., Hofacker I.L. (2007). RNA maps reveal new RNA classes and a possible function for pervasive transcription. Science.

[22] Efroni S., Duttagupta R., Cheng J., Dehghani H., Hoeppner D.J., Dash C., Bazett-Jones D.P., Le Grice S., McKay R.D., Buetow K.H. (2008). Global transcription in pluripotent embryonic stem cells. Cell Stem Cell.

[23] Clark M.B., Amaral P.P., Schlesinger F.J., Dinger M.E., Taft R.J., Rinn J.L., Ponting C.P., Stadler P.F., Morris K.V., Morillon A. (2011). The reality of pervasive transcription. PLoS Biol.

[24] St Laurent G., Shtokalo D., Tackett M.R., Yang Z., Eremina T., Wahlestedt C., Urcuqui-Inchima S., Seilheimer B., McCaffrey T.A., Kapranov P. (2012). Intronic RNAs constitute the major fraction of the non-coding RNA in mammalian cells. BMC Genomics.

[25] Djebali S., Davis C.A., Merkel A., Dobin A., Lassmann T., Mortazavi A., Tanzer A., Lagarde J., Lin W., Schlesinger F. (2012). Landscape of transcription in human cells. Nature.

[26] Deveson I.W., Hardwick S.A., Mercer T.R., Mattick J.S. (2017). The dimensions, dynamics, and relevance of the mammalian noncoding transcriptome. Trends Genet..

[27] Deveson I.W., Brunck M.E., Blackburn J., Tseng E., Hon T., Clark T.A., Clark M.B., Crawford J., Dinger M.E., Nielsen L.K. (2018). Universal alternative splicing of noncoding exons. Cell Syst..

[28] Liu G., Mattick J.S., Taft R.J. (2013). A meta-analysis of the genomic and transcriptomic composition of complex life. Cell Cycle.

[29] Faulkner G.J., Kimura Y., Daub C.O., Wani S., Plessy C., Irvine K.M., Schroder K., Cloonan N., Steptoe A.L., Lassmann T. (2009). The regulated retrotransposon transcriptome of mammalian cells. Nat. Genet..

[30] Fort A., Hashimoto K., Yamada D., Salimullah M., Keya C.A., Saxena A., Bonetti A., Voineagu I., Bertin N., Kratz A. (2014). Deep transcriptome profiling of mammalian stem cells supports a regulatory role for retrotransposons in pluripotency maintenance. Nat. Genet..

[31] Fejes-Toth K., Sotirova V., Sachidanandam R., Assaf G., Hannon G.J., Kapranov P., Foissac S., Willingham A.T., Duttagupta R., Dumais E. (2009). Post-transcriptional processing generates a diversity of 5′-modified long and short RNAs. Nature.

[32] Mercer T.R., Dinger M.E., Bracken C.P., Kolle G., Szubert J.M., Korbie D.J., Askarian-Amiri M.E., Gardiner B.B., Goodall G.J., Grimmond S.M. (2010). Regulated post-transcriptional RNA cleavage diversifies the eukaryotic transcriptome. Genome Res..

[33] Burd C.E., Jeck W.R., Liu Y., Sanoff H.K., Wang Z., Sharpless N.E. (2010). Expression of linear and novel circular forms of an *INK4/ARF*-associated non-coding RNA correlates with atherosclerosis risk. PLoS Genet..

[34] Zhang Y., Zhang X.O., Chen T., Xiang J.F., Yin Q.F., Xing Y.H., Zhu S., Yang L., Chen L.L. (2013). Circular intronic long noncoding RNAs. Mol. Cell.

[35] Furuno M., Pang K.C., Ninomiya N., Fukuda S., Frith M.C., Bult C., Kai C., Kawai J., Carninci P., Hayashizaki Y. (2006). Clusters of internally primed transcripts reveal novel long noncoding RNAs. PLoS Genet..

[36] St Laurent G., Shtokalo D., Dong B., Tackett M.R., Fan X., Lazorthes S., Nicolas E., Sang N., Triche T.J., McCaffrey T.A. (2013). VlincRNAs controlled by retroviral elements are a hallmark of pluripotency and cancer. Genome Biol..

[37] Mattick J.S. (1994). Introns: Evolution and function. Curr. Opin. Genet. Dev..

[38] Mattick J.S. (2001). Non-coding RNAs: The architects of eukaryotic complexity. EMBO Rep..

[39] Mattick J.S. (2004). RNA regulation: A new genetics?. Nat. Rev. Genet..

[40] Ohno S. (1972). So much “junk” DNA in our genome. Brookhaven Symp. Biol..

[41] Gilbert W. (1978). Why genes in pieces?. Nature.

[42] Orgel L.E., Crick F.H. (1980). Selfish DNA: The ultimate parasite. Nature.

[43] Gutschner T., Hammerle M., Eissmann M., Hsu J., Kim Y., Hung G., Revenko A., Arun G., Stentrup M., Gross M. (2013). The noncoding RNA MALAT1 is a critical regulator of the metastasis phenotype of lung cancer cells. Cancer Res..

[44] Waterston R.H., Lindblad-Toh K., Birney E., Rogers J., Abril J.F., Agarwal P., Agarwala R., Ainscough R., Alexandersson M., An P. (2002). Initial sequencing and comparative analysis of the mouse genome. Nature.

[45] Pheasant M., Mattick J.S. (2007). Raising the estimate of functional human sequences. Genome Res..

[46] Hasler J., Samuelsson T., Strub K. (2007). Useful ‘junk’: *Alu* RNAs in the human transcriptome. Cell Mol. Life Sci..

[47] Cordaux R., Batzer M.A. (2009). The impact of retrotransposons on human genome evolution. Nat. Rev. Genet..

[48] Faulkner G.J., Carninci P. (2009). Altruistic functions for selfish DNA. Cell Cycle.

[49] Johnson R., Guigo R. (2014). The RIDL hypothesis: Transposable elements as functional domains of long noncoding RNAs. RNA.

[50] Smith N.G., Brandstrom M., Ellegren H. (2004). Evidence for turnover of functional noncoding DNA in mammalian genome evolution. Genomics.

[51] Pang K.C., Frith M.C., Mattick J.S. (2006). Rapid evolution of noncoding RNAs: Lack of conservation does not mean lack of function. Trends Genet..

[52] Taft R.J., Pheasant M., Mattick J.S. (2007). The relationship between non-protein-coding DNA and eukaryotic complexity. Bioessays.

[53] Oldmeadow C., Mengersen K., Mattick J.S., Keith J.M. (2010). Multiple evolutionary rate classes in animal genome evolution. Mol. Biol. Evol..

[54] Smith M.A., Gesell T., Stadler P.F., Mattick J.S. (2013). Widespread purifying selection on RNA structure in mammals. Nucleic Acids Res..

[55] Mattick J.S. (2009). The genetic signatures of noncoding RNAs. PLoS Genet..

[56] Dinger M.E., Amaral P.P., Mercer T.R., Mattick J.S. (2009). Pervasive transcription of the eukaryotic genome: Functional indices and conceptual implications. Brief Funct. Genomic. Proteomic..

[57] Derrien T., Johnson R., Bussotti G., Tanzer A., Djebali S., Tilgner H., Guernec G., Martin D., Merkel A., Knowles D.G. (2012). The GENCODE v7 catalog of human long noncoding RNAs: Analysis of their gene structure, evolution, and expression. Genome Res..

[58] Cawley S., Bekiranov S., Ng H.H., Kapranov P., Sekinger E.A., Kampa D., Piccolboni A., Sementchenko V., Cheng J., Williams A.J. (2004). Unbiased mapping of transcription factor binding sites along human chromosomes 21 and 22 points to widespread regulation of noncoding RNAs. Cell.

[59] Guttman M., Amit I., Garber M., French C., Lin M.F., Feldser D., Huarte M., Zuk O., Carey B.W., Cassady J.P. (2009). Chromatin signature reveals over a thousand highly conserved large non-coding RNAs in mammals. Nature.

[60] Ravasi T., Suzuki H., Pang K.C., Katayama S., Furuno M., Okunishi R., Fukuda S., Ru K., Frith M.C., Gongora M.M. (2006). Experimental validation of the regulated expression of large numbers of non-coding RNAs from the mouse genome. Genome Res..

[61] Dinger M.E., Amaral P.P., Mercer T.R., Pang K.C., Bruce S.J., Gardiner B.B., Askarian-Amiri M.E., Ru K., Solda G., Simons C. (2008). Long noncoding RNAs in mouse embryonic stem cell pluripotency and differentiation. Genome Res..

[62] Pang K.C., Dinger M.E., Mercer T.R., Malquori L., Grimmond S.M., Chen W., Mattick J.S. (2009). Genome-wide identification of long noncoding RNAs in CD8^+^ T cells. J. Immunol..

[63] Mercer T.R., Dinger M.E., Sunkin S.M., Mehler M.F., Mattick J.S. (2008). Specific expression of long noncoding RNAs in the mouse brain. Proc. Natl. Acad. Sci. USA.

[64] Mercer T.R., Qureshi I.A., Gokhan S., Dinger M.E., Li G., Mattick J.S., Mehler M.F. (2010). Long noncoding RNAs in neuronal-glial fate specification and oligodendrocyte lineage maturation. BMC Neurosci..

[65] Mercer T.R., Gerhardt D.J., Dinger M.E., Crawford J., Trapnell C., Jeddeloh J.A., Mattick J.S., Rinn J.L. (2012). Targeted RNA sequencing reveals the deep complexity of the human transcriptome. Nat. Biotechnol..

[66] Mercer T.R., Wilhelm D., Dinger M.E., Solda G., Korbie D.J., Glazov E.A., Truong V., Schwenke M., Simons C., Matthaei K.I. (2011). Expression of distinct RNAs from 3′ untranslated regions. Nucleic Acids Res..

[67] Kocabas A., Duarte T., Kumar S., Hynes M.A. (2015). Widespread differential expression of coding region and 3′ UTR sequences in neurons and other tissues. Neuron.

[68] Amaral P.P., Clark M.B., Gascoigne D.K., Dinger M.E., Mattick J.S. (2011). lncRNAdb: A reference database for long noncoding RNAs. Nucleic Acids Res..

[69] Quek X.C., Thomson D.W., Maag J.L., Bartonicek N., Signal B., Clark M.B., Gloss B.S., Dinger M.E. (2015). lncRNAdb v2.0: Expanding the reference database for functional long noncoding RNAs. Nucleic Acids Res..

[70] Volders P.J., Verheggen K., Menschaert G., Vandepoele K., Martens L., Vandesompele J., Mestdagh P. (2015). An update on LNCipedia: A database for annotated human lncRNA sequences. Nucleic Acids Res..

[71] Fang S., Zhang L., Guo J., Niu Y., Wu Y., Li H., Zhao L., Li X., Teng X., Sun X. (2018). NONCODEV5: A comprehensive annotation database for long non-coding RNAs. Nucleic Acids Res..

[72] Sunwoo H., Dinger M.E., Wilusz J.E., Amaral P.P., Mattick J.S., Spector D.L. (2009). MEN ε/β nuclear-retained non-coding RNAs are up-regulated upon muscle differentiation and are essential components of paraspeckles. Genome Res..

[73] Bond C.S., Fox A.H. (2009). Paraspeckles: Nuclear bodies built on long noncoding RNA. J. Cell Biol..

[74] Sone M., Hayashi T., Tarui H., Agata K., Takeichi M., Nakagawa S. (2007). The mRNA-like noncoding RNA Gomafu constitutes a novel nuclear domain in a subset of neurons. J. Cell Sci..

[75] Rinn J.L., Kertesz M., Wang J.K., Squazzo S.L., Xu X., Brugmann S.A., Goodnough L.H., Helms J.A., Farnham P.J., Segal E. (2007). Functional demarcation of active and silent chromatin domains in human HOX loci by noncoding RNAs. Cell.

[76] Romero-Barrios N., Legascue M.F., Benhamed M., Ariel F., Crespi M. (2018). Splicing regulation by long noncoding RNAs. Nucleic Acids Res..

[77] Gonzalez I., Munita R., Agirre E., Dittmer T.A., Gysling K., Misteli T., Luco R.F. (2015). A lncRNA regulates alternative splicing via establishment of a splicing-specific chromatin signature. Nat. Struct. Mol. Biol..

[78] Kim T.K., Hemberg M., Gray J.M., Costa A.M., Bear D.M., Wu J., Harmin D.A., Laptewicz M., Barbara-Haley K., Kuersten S. (2010). Widespread transcription at neuronal activity-regulated enhancers. Nature.

[79] Ørom U.A., Derrien T., Beringer M., Gumireddy K., Gardini A., Bussotti G., Lai F., Zytnicki M., Notredame C., Huang Q. (2010). Long noncoding RNAs with enhancer-like function in human cells. Cell.

[80] Orom U.A., Shiekhattar R. (2013). Long noncoding RNAs usher in a new era in the biology of enhancers. Cell.

[81] Pandey R.R., Mondal T., Mohammad F., Enroth S., Redrup L., Komorowski J., Nagano T., Mancini-Dinardo D., Kanduri C. (2008). *Kcnq1ot1* antisense noncoding RNA mediates lineage-specific transcriptional silencing through chromatin-level regulation. Mol. Cell.

[82] Tsai M.C., Manor O., Wan Y., Mosammaparast N., Wang J.K., Lan F., Shi Y., Segal E., Chang H.Y. (2010). Long noncoding RNA as modular scaffold of histone modification complexes. Science.

[83] Kotake Y., Nakagawa T., Kitagawa K., Suzuki S., Liu N., Kitagawa M., Xiong Y. (2011). Long non-coding RNA *ANRIL* is required for the *PRC2* recruitment to and silencing of p15(INK4B) tumor suppressor gene. Oncogene.

[84] Khalil A.M., Guttman M., Huarte M., Garber M., Raj A., Rivea Morales D., Thomas K., Presser A., Bernstein B.E., van Oudenaarden A. (2009). Many human large intergenic noncoding RNAs associate with chromatin-modifying complexes and affect gene expression. Proc. Natl. Acad. Sci. USA.

[85] Zhang H., Zeitz M.J., Wang H., Niu B., Ge S., Li W., Cui J., Wang G., Qian G., Higgins M.J. (2014). Long noncoding RNA-mediated intrachromosomal interactions promote imprinting at the *Kcnq1* locus. J. Cell Biol..

[86] Mohammad F., Mondal T., Guseva N., Pandey G.K., Kanduri C. (2010). *Kcnq1ot1* noncoding RNA mediates transcriptional gene silencing by interacting with *Dnmt1*. Development.

[87] Di Ruscio A., Ebralidze A.K., Benoukraf T., Amabile G., Goff L.A., Terragni J., Figueroa M.E., De Figueiredo Pontes L.L., Alberich-Jorda M., Zhang P. (2013). *DNMT1*-interacting RNAs block gene-specific DNA methylation. Nature.

[88] Joung J., Engreitz J.M., Konermann S., Abudayyeh O.O., Verdine V.K., Aguet F., Gootenberg J.S., Sanjana N.E., Wright J.B., Fulco C.P. (2017). Genome-scale activation screen identifies a lncRNA locus regulating a gene neighbourhood. Nature.

[89] Anderson K.M., Anderson D.M., McAnally J.R., Shelton J.M., Bassel-Duby R., Olson E.N. (2016). Transcription of the non-coding RNA upperhand controls *Hand2* expression and heart development. Nature.

[90] Furlan G., Gutierrez Hernandez N., Huret C., Galupa R., van Bemmel J.G., Romito A., Heard E., Morey C., Rougeulle C. (2018). The *Ftx* noncoding locus controls X chromosome inactivation independently of its RNA products. Mol. Cell.

[91] Cho S.W., Xu J., Sun R., Mumbach M.R., Carter A.C., Chen Y.G., Yost K.E., Kim J., He J., Nevins S.A. (2018). Promoter of lncRNA gene PVT1 is a tumor-suppressor DNA boundary element. Cell.

[92] Amaral P.P., Mattick J.S. (2008). Noncoding RNA in development. Mamm. Genome.

[93] St Laurent G., Vyatkin Y., Antonets D., Ri M., Qi Y., Saik O., Shtokalo D., de Hoon M.J., Kawaji H., Itoh M. (2016). Functional annotation of the vlinc class of non-coding RNAs using systems biology approach. Nucleic Acids Res..

[94] Broadbent H.M., Peden J.F., Lorkowski S., Goel A., Ongen H., Green F., Clarke R., Collins R., Franzosi M.G., Tognoni G. (2008). Susceptibility to coronary artery disease and diabetes is encoded by distinct, tightly linked SNPs in the ANRIL locus on chromosome 9p. Hum. Mol. Genet..

[95] Pasmant E., Sabbagh A., Vidaud M., Bieche I. (2011). ANRIL, a long, noncoding RNA, is an unexpected major hotspot in GWAS. FASEB J..

[96] Barry G., Briggs J.A., Vanichkina D.P., Poth E.M., Beveridge N.J., Ratnu V.S., Nayler S.P., Nones K., Hu J., Bredy T.W. (2014). The long non-coding RNA Gomafu is acutely regulated in response to neuronal activation and involved in schizophrenia-associated alternative splicing. Mol. Psychiatry.

[97] Mourtada-Maarabouni M., Pickard M.R., Hedge V.L., Farzaneh F., Williams G.T. (2009). GAS5, a non-protein-coding RNA, controls apoptosis and is downregulated in breast cancer. Oncogene.

[98] Liu P.Y., Erriquez D., Marshall G.M., Tee A.E., Polly P., Wong M., Liu B., Bell J.L., Zhang X.D., Milazzo G. (2014). Effects of a novel long noncoding RNA, lncUSMycN, on N-*Myc* expression and neuroblastoma progression. J. Natl. Cancer Inst..

[99] Sauvageau M., Goff L.A., Lodato S., Bonev B., Groff A.F., Gerhardinger C., Sanchez-Gomez D.B., Hacisuleyman E., Li E., Spence M. (2013). Multiple knockout mouse models reveal lincRNAs are required for life and brain development. Elife.

[100] Mattick J.S. (2013). Probing the phenomics of noncoding RNA. Elife.

[101] Skryabin B.V., Sukonina V., Jordan U., Lewejohann L., Sachser N., Muslimov I., Tiedge H., Brosius J. (2003). Neuronal untranslated *BC1* RNA: Targeted gene elimination in mice. Mol. Cell. Biol..

[102] Lewejohann L., Skryabin B.V., Sachser N., Prehn C., Heiduschka P., Thanos S., Jordan U., Dell’Omo G., Vyssotski A.L., Pleskacheva M.G. (2004). Role of a neuronal small non-messenger RNA: Behavioural alterations in *BC1* RNA-deleted mice. Behav. Brain Res..

[103] Bejerano G., Pheasant M., Makunin I., Stephen S., Kent W.J., Mattick J.S., Haussler D. (2004). Ultraconserved elements in the human genome. Science.

[104] Stephen S., Pheasant M., Makunin I.V., Mattick J.S. (2008). Large-scale appearance of ultraconserved elements in tetrapod genomes and slowdown of the molecular clock. Mol. Biol. Evol..

[105] Ahituv N., Zhu Y., Visel A., Holt A., Afzal V., Pennacchio L.A., Rubin E.M. (2007). Deletion of ultraconserved elements yields viable mice. PLoS Biol..

[106] Dickel D.E., Ypsilanti A.R., Pla R., Zhu Y., Barozzi I., Mannion B.J., Khin Y.S., Fukuda-Yuzawa Y., Plajzer-Frick I., Pickle C.S. (2018). Ultraconserved enhancers are required for normal development. Cell.

[107] Mattick J.S., Gagen M.J. (2001). The evolution of controlled multitasked gene networks: The role of introns and other noncoding RNAs in the development of complex organisms. Mol. Biol. Evol..

[108] Mattick J.S. (2003). Challenging the dogma: The hidden layer of non-protein-coding RNAs in complex organisms. Bioessays.

[109] Mattick J.S. (2007). A new paradigm for developmental biology. J. Exp. Biol..

[110] Amaral P.P., Dinger M.E., Mercer T.R., Mattick J.S. (2008). The eukaryotic genome as an RNA machine. Science.

[111] Mattick J.S. (2011). The central role of RNA in human development and cognition. FEBS Lett..

[112] Morris K.V., Mattick J.S. (2014). The rise of regulatory RNA. Nat. Rev. Genet..

[113] Pollard K.S., Salama S.R., Lambert N., Lambot M.A., Coppens S., Pedersen J.S., Katzman S., King B., Onodera C., Siepel A. (2006). An RNA gene expressed during cortical development evolved rapidly in humans. Nature.

[114] Necsulea A., Soumillon M., Warnefors M., Liechti A., Daish T., Zeller U., Baker J.C., Grutzner F., Kaessmann H. (2014). The evolution of lncRNA repertoires and expression patterns in tetrapods. Nature.

[115] Mehler M.F., Mattick J.S. (2007). Noncoding RNAs and RNA editing in brain development, functional diversification, and neurological disease. Physiol. Rev..

[116] Mercer T.R., Dinger M.E., Mariani J., Kosik K.S., Mehler M.F., Mattick J.S. (2008). Noncoding RNAs in long-term memory formation. Neuroscientist.

[117] Barry G., Mattick J.S. (2012). The role of regulatory RNA in cognitive evolution. Trends Cogn. Sci..

[118] Mattick J.S. (2009). Deconstructing the dogma: A new view of the evolution and genetic programming of complex organisms. Ann. N. Y. Acad. Sci..

[119] Mattick J.S., Taft R.J., Faulkner G.J. (2010). A global view of genomic information--moving beyond the gene and the master regulator. Trends Genet..

[120] Mercer T.R., Mattick J.S. (2013). Structure and function of long noncoding RNAs in epigenetic regulation. Nat. Struct. Mol. Biol..

[121] Smith M.A., Seemann S.E., Quek X.C., Mattick J.S. (2017). DotAligner: Identification and clustering of RNA structure motifs. Genome Biol..

[122] Kalvari I., Nawrocki E.P., Argasinska J., Quinones-Olvera N., Finn R.D., Bateman A., Petrov A.I. (2018). Non-coding RNA analysis using the Rfam database. Curr. Protoc. Bioinformatics.

[123] Kalvari I., Argasinska J., Quinones-Olvera N., Nawrocki E.P., Rivas E., Eddy S.R., Bateman A., Finn R.D., Petrov A.I. (2018). Rfam 13.0: Shifting to a genome-centric resource for non-coding RNA families. Nucleic Acids Res..

[124] Boccaletto P., Magnus M., Almeida C., Zyla A., Astha A., Pluta R., Baginski B., Jankowska E., Dunin-Horkawicz S., Wirecki T.K. (2018). RNArchitecture: A database and a classification system of RNA families, with a focus on structural information. Nucleic Acids Res..

[125] Hentze M.W., Castello A., Schwarzl T., Preiss T. (2018). A brave new world of RNA-binding proteins. Nat. Rev. Mol. Cell Biol..

[126] Yablonovitch A.L., Deng P., Jacobson D., Li J.B. (2017). The evolution and adaptation of A-to-I RNA editing. PLoS Genet..

[127] Eisenberg E., Levanon E.Y. (2018). A-to-I RNA editing—Immune protector and transcriptome diversifier. Nat. Rev. Genet..

[128] Jonkhout N., Tran J., Smith M.A., Schonrock N., Mattick J.S., Novoa E.M. (2017). The RNA modification landscape in human disease. RNA.

[129] Novoa E.M., Mason C.E., Mattick J.S. (2017). Charting the unknown epitranscriptome. Nat. Rev. Mol. Cell Biol..

[130] Mattick J.S. (2010). RNA as the substrate for epigenome-environment interactions: RNA guidance of epigenetic processes and the expansion of RNA editing in animals underpins development, phenotypic plasticity, learning, and cognition. Bioessays.

[131] Taft R.J., Glazov E.A., Cloonan N., Simons C., Stephen S., Faulkner G.J., Lassmann T., Forrest A.R., Grimmond S.M., Schroder K. (2009). Tiny RNAs associated with transcription start sites in animals. Nat. Genet..

[132] Taft R.J., Simons C., Nahkuri S., Oey H., Korbie D.J., Mercer T.R., Holst J., Ritchie W., Wong J.J., Rasko J.E. (2010). Nuclear-localized tiny RNAs are associated with transcription initiation and splice sites in metazoans. Nat. Struct. Mol. Biol..

[133] Haussecker D., Huang Y., Lau A., Parameswaran P., Fire A.Z., Kay M.A. (2010). Human tRNA-derived small RNAs in the global regulation of RNA silencing. RNA.

[134] Zhu L., Liu X., Pu W., Peng Y. (2018). tRNA-derived small non-coding RNAs in human disease. Cancer Lett..

[135] Guzzi N., Ciesla M., Ngoc P.C.T., Lang S., Arora S., Dimitriou M., Pimkova K., Sommarin M.N.E., Munita R., Lubas M. (2018). Pseudouridylation of tRNA-derived fragments steers translational control in stem cells. Cell.

[136] Taft R.J., Glazov E.A., Lassmann T., Hayashizaki Y., Carninci P., Mattick J.S. (2009). Small RNAs derived from snoRNAs. RNA.

[137] Ender C., Krek A., Friedlander M.R., Beitzinger M., Weinmann L., Chen W., Pfeffer S., Rajewsky N., Meister G. (2008). A human snoRNA with microRNA-like functions. Mol. Cell.

[138] Politz J.C., Hogan E.M., Pederson T. (2009). MicroRNAs with a nucleolar location. RNA.

[139] Scott M.S., Avolio F., Ono M., Lamond A.I., Barton G.J. (2009). Human miRNA precursors with box H/ACA snoRNA features. PLoS Comput. Biol..

[140] Herbert A., Rich A. (1999). RNA processing in evolution: The logic of soft-wired genomes. Ann. N. Y. Acad. Sci..

[141] Herbert A., Rich A. (1999). RNA processing and the evolution of eukaryotes. Nature Genet..

[142] Mattick J.S. (2009). Has evolution learnt how to learn?. EMBO Rep..

[143] Rassoulzadegan M., Grandjean V., Gounon P., Vincent S., Gillot I., Cuzin F. (2006). RNA-mediated non-mendelian inheritance of an epigenetic change in the mouse. Nature.

[144] Dinger M.E., Mercer T.R., Mattick J.S. (2008). RNAs as extracellular signaling molecules. J. Mol. Endocrinol..

[145] Liebers R., Rassoulzadegan M., Lyko F. (2014). Epigenetic regulation by heritable RNA. PLoS Genet..

[146] Benito E., Kerimoglu C., Ramachandran B., Pena-Centeno T., Jain G., Stilling R.M., Islam M.R., Capece V., Zhou Q., Edbauer D. (2018). RNA-dependent intergenerational inheritance of enhanced synaptic plasticity after environmental enrichment. Cell Rep..

[147] Neeb Z.T., Nowacki M. (2018). RNA-mediated transgenerational inheritance in ciliates and plants. Chromosoma.

[148] Rechavi O., Lev I. (2017). Principles of transgenerational small RNA inheritance in *Caenorhabditis elegans*. Curr. Biol..

[149] Ghanbarian H., Wagner N., Michiels J.F., Cuzin F., Wagner K.D., Rassoulzadegan M. (2017). Small RNA-directed epigenetic programming of embryonic stem cell cardiac differentiation. Sci. Rep..

